# Racial Differences in Vascular Assessment Prior to Amputation in the Veterans Health Administration

**DOI:** 10.1089/heq.2023.0004

**Published:** 2023-05-26

**Authors:** Olamide Alabi, Kelly J. Hunt, Rachel E. Patzer, Tabia Henry Akintobi, Nader N. Massarweh

**Affiliations:** ^1^ Surgical and Perioperative Care, Atlanta VA Health Care System, Decatur, Georgia, USA.; ^2^ Division of Vascular Surgery, Department of Surgery, Emory University School of Medicine, Atlanta, Georgia, USA.; ^3^ Charleston Health Equity and Rural Outreach Innovation Center (HEROIC), Ralph H. Johnson VA Medical Center, Charleston, South Carolina, USA.; ^4^ Department of Public Health Sciences, Medical University of South Carolina, Charleston, South Carolina, USA.; ^5^ Health Services Research Center, Emory University School of Medicine, Atlanta, Georgia, USA.; ^6^ Department of Community Health and Preventive Medicine, Morehouse School of Medicine, Atlanta, Georgia, USA.; ^7^ Division of Surgical Oncology, Department of Surgery, Emory University School of Medicine, Atlanta, Georgia, USA.; ^8^ Department of Surgery, Morehouse School of Medicine, Atlanta, Georgia, USA.

**Keywords:** peripheral artery disease, amputation, racial disparities, vascular assessment, quality of care

## Abstract

**Purpose:**

It is unclear whether disparities in the care provided before lower extremity amputation (LEA) is driven by differences in receipt of diagnostic work-up versus revascularization attempts.

**Methods:**

We performed a national cohort study of Veterans who underwent LEA between March 2010 and February 2020 to assess receipt of vascular assessment with arterial imaging and/or revascularization in the year prior to LEA.

**Results:**

Among 19,396 veterans (mean age 66.8 years; 26.6% Black), Black veterans had diagnostic procedures more often than White veterans (47.5% vs. 44.5%) and revascularization as often (25.8% vs. 24.5%).

**Conclusion:**

We must identify patient and facility-level factors associated with LEA as disparities do not appear related to differences in attempted revascularization.

## Introduction

Over 12 million Americans have peripheral artery disease (PAD), a condition wherein insufficient blood flow to the lower extremities can cause poor function, nonhealing wounds, or gangrene. PAD can lead to significant morbidity and cardiovascular mortality.^1^ In fact, PAD is one of the leading causes of major lower extremity amputation (LEA) with up to 25% of patients eventually requiring amputation within a year after a new diagnosis of severe PAD.^2^ Rates of major amputation among individuals from minority racial and ethnic communities range between two and four times that of White patients.^3–8^

It is estimated that nearly 10% of all veterans have PAD, and ∼1800 veterans undergo amputation each year within the Veterans Health Administration (VHA). Most amputations can be prevented with well-established diagnostic and therapeutic interventions; however, utilization of these strategies is variable.^9^

Several studies among nonveterans report significantly low rates of diagnostic vascular assessment as well as attempts at revascularization before LEA. Goodney et al. reported that 54% of Medicare beneficiaries do not have an attempt at revascularization before an amputation.^10^ It is currently unknown why vascular assessment and attempts at revascularization are not more widely used before LEA.

It is also unclear what proportion of veterans undergo only diagnostic procedures without a subsequent attempt at revascularization before LEA, and whether this may be a potential explanation for observed disparities in LEA rates. As such, the objective of this study was to determine what proportion of veterans undergo diagnostic work-up with and without an attempt at revascularization in the 1 year before LEA, and to examine whether there are racial or regional differences.

## Materials and Methods

### Study design and data sources

This study was approved by the Emory institutional review board and the Atlanta VA Health Care System research and development committee. All veterans over age 18 years who underwent LEA within the VHA between March 1, 2010, and February 28, 2020, were identified in the VA Corporate Data Warehouse. Current procedural terminology (CPT) codes for major LEA (defined as any amputation above the level of the ankle) were used to define the study population. We excluded any amputation revision and patients who received their care in the community (outside VHA).

### Outcomes

The primary outcome was the receipt of a vascular assessment in the 1 year before a major LEA. Vascular assessment was defined as any imaging study or diagnostic procedure performed on a veteran to either establish a diagnosis of arterial insufficiency (diagnostic only) or to improve lower extremity arterial flow (revascularization). We used CPT codes to determine which veterans underwent vascular assessment and whether the assessment was performed only for diagnostic purposes or was an attempt at revascularization. We excluded procedures performed for the indication of acute limb ischemia.

### Covariates

Race was the primary exposure variable of interest. We first categorized race as American Indian/Alaskan Native, Asian, Black/African American, Native Hawaiian/Pacific Islander, White, and “unknown.” However, we found that >90% of patients were either Black or White and 6% were listed as “unknown.” Therefore, we only included Black and White veterans in our analysis to compare vascular assessment among groups. Hispanic veterans were not excluded, however, analyses were not stratified by ethnicity.

Region was the secondary exposure variable of interest and was categorized as northeast, midwest, west, southwest, and southeast. This variable identified the region of the country in which the veteran resided at the time of index amputation. Age was categorized as <60, 60–69, 70–79, or ≥80 years. Body mass index (BMI) was categorized as obese (≥30 kg/m^2^) or not obese (<30 kg/m^2^). Chronic kidney disease (CKD) was categorized as no CKD, CKD (stage III and above) not on dialysis, and end-stage renal disease.^11^ All other comorbidities were defined using the International Classification of Diseases, 9th and 10th revisions (ICD-9 and ICD-10) diagnosis codes.

### Statistical analysis

Standard descriptive statistics were performed and univariate associations with the primary outcome were assessed using the odds ratio test. Categorical variables were compared using chi square analysis and Cochran–Mantel–Haenzsel test for trend was performed to assess for a difference in trend among annual volume of amputations by region. There was very little missing data (BMI, 6.1%; all other covariates, 0%). All analyses were conducted using SAS, version 9.4 (SAS Institute, Inc., Cary, NC, USA).

## Results

In total, 19,396 veterans underwent major LEA during the study period. Among them, 98.5% (*n*=12,782) were male. The mean age was 66.78 years (standard deviation, 10.2) and 64.9% (*n*=12,600) were under the age of 70 years. The cohort included 1114 (5.7%) American Indian/Alaskan Natives, 37 (0.2%) Asians, 5167 (26.6%) Black/African Americans, 133 (0.7%) Native Hawaiian/Pacific Islanders, 12,782 (65.9%) Whites, and 1114 (5.7%) veterans of unknown race. Self-identification as Hispanic ethnicity comprised 6.0% (*n*=1171) of the cohort. Demographic data for the cohort are provided in Table 1.

**Table 1.** Descriptive Statistics and Univariate Analyses Among Those Who Received Vascular Assessment Before Major Amputation

	Overall (*N*=19,396)	No vascular assessment (*n*=5836) (42.2%)	Vascular assessment (*n*=13,560) (69.9%)	Crude odds ratio (95% CI)
Age (years)				
<60	3902 (20.1)	1648 (28.2)	2254 (16.6)	Reference
60–69	8698 (44.8)	2524 (43.3)	6174 (45.5)	1.79 (1.65–1.94)
70–79	4494 (23.2)	1080 (18.5)	3414 (25.2)	2.31 (2.11–2.54)
80+	2302 (11.9)	584 (10.0)	1718 (12.7)	2.15 (1.92–2.41)
Mean (SD)	66.78 (10.2)	64.50 (11.5)	67.77 (9.4)	N/A
Birth sex				
Male	19,104 (98.5)	5700 (97.7)	13,404 (98.9)	Reference
Female	292 (1.5)	136 (2.3)	156 (1.2)	0.49 (0.39–0.62)
Race				
White	12,782 (65.9)	3962 (67.9)	8820 (65.0)	Reference
Black/African American	5167 (26.6)	1378 (23.6)	3789 (27.9)	1.24 (1.15–1.33)
Asian	37 (0.2)	16 (0.3)	21 (0.2)	0.59 (0.31–1.13)
Native Hawaiian/Pacific Islander	133 (0.7)	40 (0.7)	93 (0.7)	1.04 (0.72–1.52)
American Indian/Alaskan Native	163 (0.8)	60 (1.0)	103 (0.8)	0.77 (0.56–1.06)
Unknown	1114 (5.7)	380 (6.5)	734 (5.4)	0.87 (0.76–0.99)
Ethnicity				
Hispanic	1171 (6.0)	340 (5.8)	831 (6.1)	
Non-Hispanic	17,755 (91.5)	5326 (91.3)	12,429 (91.7)	Reference
Unknown	470 (2.4)	170 (2.9)	300 (2.2)	

CI, confidence interval; SD, standard deviation.

**FIG. 1.**
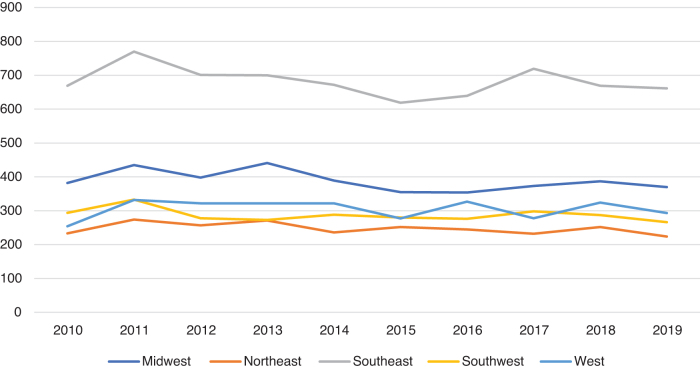
Temporal trends in amputation volume by region.

**FIG. 2.**
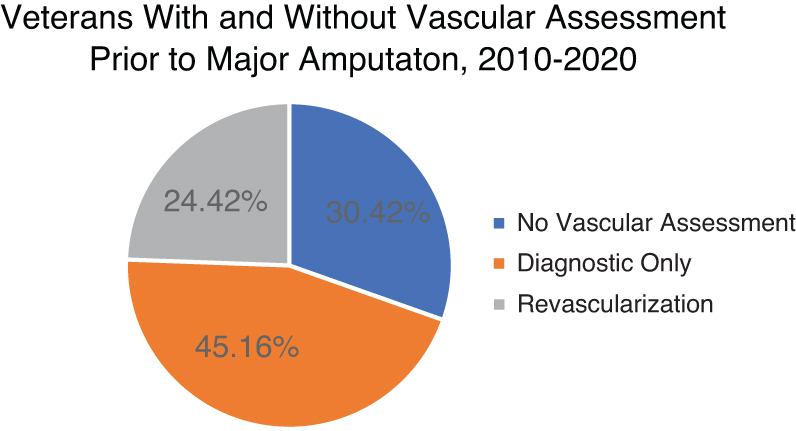
Proportion of vascular assessments performed before major amputation.

When stratified by region, the volume of major LEA did not change from year to year (*p*=0.42; Fig. 1). A vascular assessment was not performed on 5836 subjects, or 30.4% of the cohort (Fig. 2). We found that a higher proportion of Black veterans had diagnostic procedures as compared with White veterans (47.5% vs. 44.5%, *p*=0.0001). In addition, significantly more Black veterans had an attempt at revascularization than White veterans in the year before LEA (25.8% vs. 24.5%, *p*=0.03).

There were also significant regional differences noted when considering those who underwent only diagnostic procedures relative to those who underwent an attempt at revascularization (Table 2). For example, 52.9% of veterans in the midwest obtained only diagnostic imaging, while 27% had at least one attempt at revascularization in the year before LEA. Conversely, in the west, 40.8% of veterans had only diagnostic imaging performed, whereas 23.3% had an attempt at revascularization in the year before LEA.

**Table 2.** Proportion of Veterans Undergoing No Vascular Assessment, Diagnostic Only, and Revascularization in the 1 Year Before Major Amputation

	No vascular assessment	Diagnostic only	Revascularization	*p*
Race				<0.0000001
Black	1378 (26.7)	2455 (47.5)	1334 (25.8)	
White	3962 (31.0)	5692 (44.5)	3128 (24.5)	
Region				<0.0000001
Midwest	826 (21.0)	2044 (52.0)	1063 (27.0)	
Northeast	716 (28.4)	1028 (40.8)	776 (30.8)	
Southeast	2220 (32.0)	3184 (45.9)	1526 (22.0)	
Southwest	960 (33.0)	1249 (42.9)	704 (24.2)	
West	1114 (35.9)	1265 (40.8)	721 (23.3)	

## Discussion

Previously published work has demonstrated striking racial disparities in PAD outcomes—specifically, Black patients are at higher risk of major LEA than any other racial group. Many believe that most amputations are preventable when guideline-directed medical therapy and vascular assessments are performed. However, there is notable variation in who gets a vascular assessment before an LEA. In this regard, our study presents novel findings that potentially shed new light on this specific issue: (1) vascular assessment is performed in over two-thirds of veterans who undergo major LEA; (2) as compared with White veterans, Black veterans are more likely to receive diagnostic studies, as well as to have an attempt at revascularization before LEA.

Over the last few decades, Black patients have consistently been found to have higher LEA rates than White patients.^3–6^ There are several conflicting ideas as to why this may be the case, and very few proposed solutions to address or eliminate this disparity.^8,12–14^ Some have suggested Black patients may have a lower likelihood of being offered revascularization, which in turn leads to higher LEA rates.^4,15,16^ In our national cohort of veterans, Black patients represented over one-quarter of the study population (this over-represents this subgroup relative to the U.S. population and the veteran population receiving care at the VA).^17^

Despite this over-representation, our work suggests Black veterans receive both diagnostic studies and attempts at revascularization more often than White veterans. As such, it does not appear that a lower likelihood of attempted revascularization is a major driver of racial disparities in LEA, at least among veterans receiving care at the VHA. Further evaluation of clinical indications for LEA, exploration of health care utilization among Veterans, and examination of prescription of guideline concordant care among Veteran providers is needed.

There are some limitations of note that should be considered. This was an observational study of a cohort of veterans who underwent major LEA. As such, selection bias cannot be entirely excluded as a reason for our study findings. As well, we did not perform a deep dive into veteran comorbidities in this study and their association with components of vascular assessment: diagnostic and therapeutic interventions. It is well known that diabetes mellitus and severe renal disease each are risk factors for PAD that work synergistically to elevate the risk for LEA.^18,19^

In addition, because the data were abstracted from the VA Corporate Data Warehouse, we did not include veterans who may have undergone an LEA outside of VHA. As such, we may have underestimated the proportion of veterans who receive vascular assessment before LEA, as we did not include data from care in the private sector. Finally, it is unclear how severe a patient's PAD was on presentation or whether some LEAs were performed in an emergent setting due to severe infection.

## Conclusions

Although racial disparities in major LEAs exist within the VHA, our findings suggest this does not appear to be due to racial differences in the receipt of diagnostic or revascularization procedures within the VHA. Future study should consider patient and facility factors that may be associated with receipt of vascular assessment before a major LEA.

## Abbreviations Used

CI: confidence interval
CPT: current procedural terminology
ICD-9: International Classification of Diseases, 9th revision
ICD-10: International Classification of Diseases, 10th revision
LEA: lower extremity amputation
PAD: peripheral artery disease
SD: standard deviation
VHA: Veterans Health Administration

## References

[1] Fowkes GF, Rudan D, Rudan I, et al. Comparison of global estimates of prevalence and risk factors for peripheral artery disease in 2000 and 2010: A systematic review and analysis. Lancet 2013;382:1329–1340. 23915883 10.1016/S0140-6736(13)61249-0

[2] Mustapha J, Katzen BT, Neville RF, et al. Determinants of long-term outcomes and costs in the management of critical limb ischemia: A population-based cohort study. J Am Heart Assoc 2018;7(16):e009724. 30369325 10.1161/JAHA.118.009724 PMC6201392

[3] Arya S, Binney Z, Khakharia A, et al. Race and socioeconomic status independently affect risk of major amputation in peripheral artery disease. J Am Heart Assoc 2018;7(2):e007425. 29330260 10.1161/JAHA.117.007425 PMC5850162

[4] Holman KH, Henke PK, Dimick JB, et al. Racial disparities in the use of revascularization before leg amputation in Medicare patients. J Vasc Surg 2011;54(2):420–426.e1. 21571495 10.1016/j.jvs.2011.02.035 PMC3152619

[5] Eslami MH, Zayaruzny M, Fitzgerald GA. The adverse effects of race, insurance status, and low income on the rate of amputation in patients presenting with lower extremity ischemia. J Vasc Surg 2007;45(1):55–59. 17210382 10.1016/j.jvs.2006.09.044

[6] Durazzo TS, Frencher S, Gusberg R. Influence of race on the management of lower extremity ischemia: Revascularization vs amputation. JAMA Surg 2013;148(7):617–623. 23552850 10.1001/jamasurg.2013.1436

[7] Collins TC, Johnson M, Henderson W, et al. Lower extremity nontraumatic amputation among veterans with peripheral arterial disease: Is race an independent factor? Med Care 2002;40(1 Suppl):I106–I116. 11789623 10.1097/00005650-200201001-00012

[8] Rivero M, Nader ND, Blochle R, et al. Poorer limb salvage in African American men with chronic limb ischemia is due to advanced clinical stage and higher anatomic complexity at presentation. J Vasc Surg 2016;63(5):1318–1324. 27005751 10.1016/j.jvs.2015.11.052

[9] Creager MA, Matsushita K, Arya S, et al. Reducing nontraumatic lower-extremity amputations by 20% by 2030: Time to get to our feet: A policy statement from the American Heart Association. Circulation 2021;143(17):e875–e891. 33761757 10.1161/CIR.0000000000000967

[10] Goodney PP, Travis LL, Nallamothu BK, et al. Variation in the use of lower extremity vascular procedures for critical limb ischemia. Circ Cardiovasc Qual Outcomes 2012;5(1):94–102. 22147886 10.1161/CIRCOUTCOMES.111.962233 PMC3281555

[11] Kidney Disease: Improving Global Outcomes Chronic Kidney Disease Guideline Development Work Group Members. KDIGO 2012 clinical practice guideline for the evaluation and management of chronic kidney disease. Kidney Int Suppl 2013;3:1–150.

[12] Sidawy AN, Schweitzer EJ, Neville RF, et al. Race as a risk factor in the severity of infragenicular occlusive disease: Study of an urban hospital patient population. J Vasc Surg 1990;11(4):536–543. 2325214

[13] Mustapha JA, Fisher BT, Sr., Rizzo JA, et al. Explaining racial disparities in amputation rates for the Treatment of Peripheral Artery Disease (PAD) using decomposition methods. J Racial Ethn Health Disparities 2017;4(5):784–795. 28205152 10.1007/s40615-016-0261-9 PMC5626799

[14] Kalbaugh CA, Witrick B, Sivaraj LB, et al. Non-Hispanic Black and Hispanic patients have worse outcomes than white patients within similar stages of peripheral artery disease. J Am Heart Assoc 2022;11(1):e023396. 34927446 10.1161/JAHA.121.023396 PMC9075215

[15] Regenbogen SE, Gawande AA, Lipsitz SR, et al. Do differences in hospital and surgeon quality explain racial disparities in lower-extremity vascular amputations? Ann Surg 2009;250(3):424–431. 19652590 10.1097/SLA.0b013e3181b41d53

[16] Goodney PP, Holman K, Henke PK, et al. Regional intensity of vascular care and lower extremity amputation rates. J Vasc Surg 2013;57(6):1471–1479, 1480.e1–e3; discussion 9–80. 23375611 10.1016/j.jvs.2012.11.068 PMC3660510

[17] VA Office of Health Equity. National Veteran Health Equity Report-FY2013. US Department of Veterans Affairs: Washington, DC, USA; 2016.

[18] O'Hare A, Johansen K. Lower-extremity peripheral arterial disease among patients with end-stage renal disease. J Am Soc Nephrol 2001;12(12):2838–2847. 11729255 10.1681/ASN.V12122838

[19] Beckman JA, Paneni F, Cosentino F, et al. Diabetes and vascular disease: Pathophysiology, clinical consequences, and medical therapy: Part II. Eur Heart J 2013;34(31):2444–2452. 23625211 10.1093/eurheartj/eht142

